# Cosmic Influence on the Sun-Earth Environment

**DOI:** 10.3390/s8127736

**Published:** 2008-12-03

**Authors:** Saumitra Mukherjee

**Affiliations:** Geology and Remote Sensing, School of Environmental Sciences, Jawaharlal Nehru University, New Delhi-110067, India; E-mail: dr.saumitramukherjee@usa.net; Tel. +91-11-26704312; Fax +91-11-26704312

**Keywords:** SOHO satellite data, cosmic, extragalactic, earthquake, change in atmosphere

## Abstract

SOHO satellite data reveals geophysical changes before sudden changes in the Earth's Sun-Earth environment. The influence of extragalactic changes on the Sun as well as the Sun-Earth environment seems to be both periodic and episodic. The periodic changes in terms of solar maxima and minima occur every 11 years, whereas the episodic changes can happen at any time. Episodic changes can be monitored by cosmic ray detectors as a sudden increase or decrease of activity. During these solar and cosmic anomaly periods the environment of the Earth is affected. The Star-Sun-Earth connection has the potential to influence the thermosphere, atmosphere, ionosphere and lithosphere. Initial correlation of the cosmic and Sun-Earth connection has shown the possibility of predicting earthquakes, sudden changes in atmospheric temperatures and erratic rainfall/snowfall patterns.

## 1. Introduction

Since the early days of human civilization we have looked at the sky and tried to understand the environment of the Earth and the Universe [1, 2, 3, 4, and 5]. We are continuously collecting data for different environmental parameters. Sudden heat or cold waves, tornados, erratic rainfall and snowfall are being observed and their forewarning has been attempted. Efforts have been made to understand the influence of stars and the Sun, which, although they are distant objects in space, can influence the environment of the Earth. Extragalactic cosmic rays measured as neutron counting rate, represent an energy spectrum, which is being received by the solar system from the distant stars; the particles of cosmic rays are atom –nuclei with almost light velocity [6, 7]. The effects of the Sun on the environment of the Earth were found to be modulated by the geomagnetic field and the ionizing potential of the cosmic rays [8]. Earth directed Coronal Mass Ejection (CME) and its effects on the thermosphere, ionosphere and atmosphere have been studied. During Earth directed CME a beam of electrons (plasma) is pumped towards the Earth [9]. This beam of electrons is highly conductive and generates an electric field that is transmitted to Earth's natural plasmosphere and ionosphere. This thin layer of changed electric field further influences the ionosphere and atmosphere of the earth [24]. Since a beam of electrons is carried by an electric current, a magnetic disturbance would be produced. Starbursts are caused by a special variety of neutron star known as a magnetar. These fast-spinning, compact stellar bodies create intense magnetic fields that trigger explosions, which are known as starbursts. Starbursts cause the Sun to develop low Planetary Indices (Kp) and low Electron flux (E-flux) conditions for the Sun-Earth Environment.

**Figure 1.**
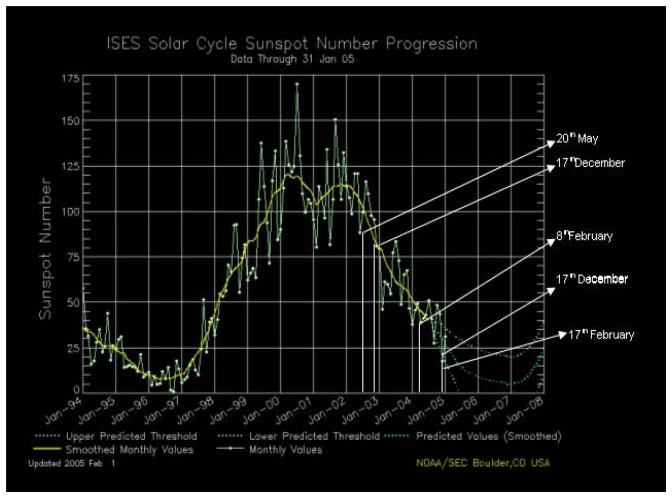
Influence of V838 Monoceras star flares (cosmic rays) on sunspot numbers. 20th May 2002, 17^th^ December 2002, 8^th^ February 2004, 17th December 2004 and 17^th^ February 2005 show low sunspot numbers and higher cosmic ray (identified by cosmic ray detector). Courtesy NASA.

E-flux induces the variation of production of ionosphere currents. Ionosphere currents are produced by geomagnetic storms originating from the Star-Sun-Earth environment. Ionosphere current variation has a direct influence on atmospheric temperature [10, 11]. On 25th December 2004 and 23rd February 2005 hailstorms and snowstorms were reported in the Northern Hemisphere, while in the Tropics a sudden drop in temperature led to foggy and smoggy conditions. This temperature variation was different in different parts of the Earth, as the effects of solar flares are dependent on the geomagnetic co-ordinate of Earth and its respective position with regards to the stars. Further, the fluctuation of atmospheric temperatures in the month of December 2005, in the first week of January 2006 and in the last week of December 2007, suggests the direct correlation of the Star-Sun-Earth environment (Figure 1). If the electron flux from the sun is low, with the subsequent rise in cosmic rays simultaneously anomalous snowfall and lowering of the atmospheric temperature has been observed. It would be possible to understand the movement of clouds and snowfall, as well as atmospheric moisture, if we could efficiently calculate the influence of space weather and cosmic influence on the thermosphere and atmosphere of the Earth [12]. Based on the same hypothesis it was found that an abnormal rise and sudden fall in E-flux, Kp index and atmospheric temperature has the possibility of triggering earthquakes in active fault areas of the Earth due to temporary changes in the magnetic field of the Earth. The whole process was expressed as a precursor of earthquakes in active fault areas.

## 2. Results and Discussion

It was not possible to explain the reason of observed solar maximum in every 11th year [13, 14 and 15]. However, the cosmic effect can reduce the development of sunspots as well as CME. A cloud with 1,000 atoms per cubic centimeter could compress the Sun's magnetic field to within a few AU of the Sun (1 AU or “one astronomical unit” is the distance between the Sun and Earth) [16, 17, 18]. Periodically, a bright object appears in a galaxy and remains that way for days to months. It is referred to, erroneously, as a new star or nova (Latin for “new”; plural “novae”). It is, in fact, a star that for more than one reason experiences a major flare-up and later dies down leaving the star intact but with loss of material. Star V838 in the Monoceras constellation underwent such a blue star flare-up, releasing considerable material as it reached a luminosity of 600,000 times that of the Sun. This made it temporarily the brightest star in our Milky Way. The light from this eruption created a unique phenomenon known as a ‘light echo’ when it reflected off dust shells around the star. This phenomenon was followed by hailstorms in the Northern Hemisphere. In the month of December 2004, the star repeated a similar phenomenon. The snowfall on the 23rd and 24th February all over England and other parts of higher altitude and latitude with low electron flux measured by SOHO satellite data confirms the hypothesis. Star flares during low Planetary Indices (Kp) and low Electron flux (E-flux) periods of the Sun-Earth environment might result in further lowering the magnetic field as well as electron flux through the repulsion of magnetic field in the Sun-Earth environment by star flares and/or other cosmic factors. The E-flux variation will in turn induce variations in the production of ionospheric currents [19, 20]. Ionosphere currents are produced by geomagnetic storms originating from the Star-Sun-Earth environment [21, 22, 23, 24 and 25]. Ionosphere current variations not only disturb radio transmissions and GPS signal reception, but it may also have an influence on atmospheric temperatures. It is a well-established fact that the formation of clouds as well as snowfall requires low temperatures as well as nucleation by dust particles [26]. The nucleation material for the snow and cloud formation is supplemented by cosmic dust particles. During low sunspot periods and with no coronal mass ejection from the Sun, the possibility of the formation of cloud and snowfall increases. To the contrary, the earth directed CME might produce an electron anomaly above the thunderstorm in the upper atmosphere.

The Sun-Earth environment has variables, which are changing on regular basis due to starbursts. These variables are the Kp, proton flux and E-flux. Sudden changes in these parameters may abruptly influence the environment of the Earth. If an E-flux hike is responsible for global warming, then an E-flux lowering may lead to snowfall, thunderstorms and erratic rainfall. A severe geomagnetic storm originating from Sunspot No. 486, located in the southern part of the Sun, on 29^th^ October 2003 has shown its effects on the environment of the Earth. It began at approximately 1,700 UT when a Coronal Mass Ejection (CME) originating from an X10-class explosion from giant sunspot 486 struck our planet's magnetic field. Proton flux, E-flux and planetary Kp indices showed a sudden rise from 17.00 UTC on 29th October. Proton flux was more than 10 4 Mev and electron flux jumped to 106 Mev, while K-indices was at a peak value of 9. The effect of this CME was observed in crustal disturbances in seismically active parts of the earth. An earthquake of 6.8 on the Richter scale was recorded off the coast of Honshu, Japan on October 31. The Honshu area of Japan is on an active fault which makes it earthquake prone [27, 28]. The triggering of the earthquake is sometimes initiated by the change in the magnetic field in Sun-Earth environment which influences the faulting further. Before the occurrence of this severe earthquake a bright aurora was seen in the sky of Japan. It is possible to correlate the triggering of the active fault in the Honshu area of Japan with the sudden change in the magnetic shield of the earth due to the impact of CME from Sun. The effect of earth directed CME would not only trigger the earthquake, but affect the whole environment of the Earth, including the destruction of ozone layers leading to climate change. Active sunspots 487, 488 and 492, along with 486 in the southern part of the Sun, continue to be a possible threat to the changes in the thermosphere, ionosphere, atmosphere and geosphere of the Earth.

The mechanism of the cosmic Sun-Earth connection on environment of the Earth has been established by correlation studies of cosmic ray variation with geophysical variables of the Sun [29, 30, 31 and 32]. The effect of Earth directed Coronal Mass Ejections (CME) from the Sun reveals a sensational impact on the atmosphere and geosphere. It has been observed that there is a close relationship between Kp values (Planetary Indices) and particle flux (Electron flux and Proton Flux) with the CME. The response of the magnetosphere to interplanetary shocks or pressure pulses can result in sudden injections of energetic particles into the inner magnetosphere. It has been recorded that 36 hours before the occurrence of an earthquake, Kp values and E-flux increase drastically. The phenomenon was recorded during the Kutch, Gujarat, earthquake of 2001. When the earth directed CME glances along the magnetic shield, local disturbances in the atmosphere of the Earth have been noticed. During the monsoon of 2003 there were continuous CME glances with the magnetic shield from a group of sunspots, which are responsible for the abnormal record rainfall in northern parts of India, especially Delhi, Himachal Pradesh and Haryana states, while states like Madhya Pradesh, Tamilnadu and other southern parts of India did not receive adequate rain. It has been discovered that the position of active sunspots and their effects on Earth co-ordinates are mirror images of each other. If the Earth directed CME impact is targeted on the magnetic shield it is responsible for a sudden increase in Kp, E-flux as well as Proton flux. The sudden increase in these parameters was found to be responsible for the change in geosphere. Contrary to this phenomenon atmospheric perturbation was preceded by the fall in Kp, E-flux and Proton flux. The Earth's magnetic field acts like a shield, protecting it from the constant stream of tiny particles ejected by the Sun, which is known as the ‘solar wind’. The solar wind itself is made up of hydrogen atoms, broken into their constituent pieces: protons and electrons. When electrons find routes into our atmosphere, they collide with and excite the atoms in the air. When these excited atoms release their energy, it is given out as light, creating the glowing ‘curtains’ we see as the Aurora Borealis (or the Aurora Australis in the Southern Hemisphere). Protons ‘stealing’ electrons from the atoms in our atmosphere cause daytime proton auroral spots. The changes in the particle flux depend on changes in magnetic and electric fields [33]. Particle flux from the Sun can suddenly change the Kp (Planetary Indices), which may be directed towards Earth in the form of magnetotail. Interaction of the CME particles with the ionosphere, Earth's upper atmosphere (between 80 and 200 km above the ground), has been noticed [34]. Scientists doing research with magnetometers just before major earthquakes have serendipitously recorded tiny, slow fluctuations in Earth's magnetic field. Satellites equipped with IR cameras could be used to detect seismic hot spots from space. In fact, when Freund and Ouzounov of the Goddard Space Flight Center (GSFC) examined infrared data collected by NASA's Terra satellite, they discovered a warming of the ground in western India just before the powerful January 26, 2001, earthquake in Gujarat.

### 2.1. Correlation of weather fluctuation with CME

To conclude these considerations let us look at most impressive demonstration of the influence of solar activity on the lower layers of our atmosphere. The new concepts concerning solar-terrestrial relationship research take into account the primary processes of the whole Sun-Earth system. Cyclic changes of the general atmosphere circulation are of prime interest as are the transformation and recurrence of circulation forms, which characterize planetary wave dynamics. The changes of the atmospheric pressure in geomagnetically and electronically excited cases (including the solar activity effect) in comparison to the variations in geomagnetically and electronically quiet cases.

Solar-atmospheric interactions were observed more in the Northern India. The Delhi, Haryana, Meghalaya Mizorum stretch of India received more than 100% of the normal rainfall during the monsoon period of 2003.

The activation of the energetically active regions occurs after the geomagnetic disturbances. The corresponding regions are Delhi-Haryana-U.P-Bihar-Bengal-Orissa-M.P-Meghalaya-Mizoram with high temperature contrasts and a vertical wind shift together with baroclinic instability.

The basic disturbances appear after the geomagnetic ones in the form of the planetary wave structures with wave numbers from 3 to 4. This corresponds to the result about the selective properties of the baroclinic instability and fits the earlier obtained results on the increase of kinetic energy of such waves after the geomagnetic disturbances. Recurrence of drought after 12 years in various parts of the Earth due to irregular rain fall is due to changes in the ionosphere triggered by the change in the Electron flux during earth directed CME.

One may conclude that the helio-geomagnetic activity comes out as an agent promoting permission of instability mechanisms in the atmosphere, liberation of useful potential energy and generation of vertical kinetic energy. The disturbances arisen in energetically active zones would be transported along the main zonal flow generating planetary waves.

### 2.2. Correlation of triggering of earthquakes with CME

In recent years, earthquake prediction has moved from being a guessing game to more of a science. The primary obstacle to understanding earthquakes was geologists' refusal to accept the concept of tectonic plate movement (continental drift). Today we know that the crust of the Earth does float on a “sea” of magma, and that earthquakes and volcanism occur primarily in those zones where one plate is rubbing against another - “fault lines”. This model is not perfect because there are often “rogue” quakes which strike in unexpected areas, but it works extraordinarily well. Nonetheless, there are certain aspects of earthquakes that lend themselves to predicting their incidence. One factor is that oscillations in the planet's magnetic field often occur right before quakes; these oscillations, as noted above, often correlate strongly with astronomical and solar events. But geologists are afraid to say something ridiculous along the lines of “sunspots cause earthquakes”, despite the fact that, in a certain sense, they do… radio wave propagation (e.g. ionospheric “whistlers”), which appears to play a role in earthquakes, and it can be studied by closely monitoring the impact of the solar wind on the ionosphere. We are interested in the sun because of the many influences it has on our lives and our environment. Beyond the obvious considerations of heat and light, some examples of these direct and indirect solar influences are the effects on short-wave radio communications, navigation, use of satellites for communication and navigation, hazards to humans and instruments in space, electrical power transmission, geomagnetic prospecting, gas pipeline monitoring, and possibly weather, seismotectonics and human and animal behavior. The Solar maximum has come again after 2000. It has been noticed that the number of sunspots are continuously on the rise. Earth directed Coronal Mass Ejections (CME) were very frequent during 2000–2002 (Figure.1). Continuous impact of the tremendous amount of energy has frequently changed the Kp (planetary indices), and free electrons in the upper part of the atmosphere. These changes in Sun-Earth environment induced by the Sun have changed the geo-sphere and atmosphere from time to time. Occurrence of earthquakes followed by CME and raised Kp values have sufficient bearing on this hypothesis. All these facts compelled us to rethink and reopen the possible researches in the Sun-Earth environment. Different types of earthquake lights have been reported before, during and after severe earthquakes. Some observers have seen red, blue or white glows, while others have described them as balls of fire or flashes from the sky. Such observations have been assigned different causes. Some attribute them to the lightning from a thundercloud; some to sparks in the electric power lines; while others to the generation of static electricity in the vicinity of the focal zone of earthquakes where relative movements of rocks may produce heat and light. Over the sea, such light could arise from luminous marine organisms excited by the vibrations produced by the earthquakes [35].

Earthquake lights have been reported before the Matsushiro swarms during the year 1965 to 1967 and before some recent earthquakes in China. During the Pattan earthquake (Pakistan) of December 1974, reliable forest officers and doctors far away from the earthquake epicenter observed earthquake lights coming from the sky. Before the occurrence of the Jabalpur earthquake (India) of 1997 a light source came from the sky before the occurrence of the earthquake [35]. Experiments have been conducted at the University of Western Ontario, London (Canada) to understand the possible mechanism of earthquake lights. It has been suggested that adsorption of condensation of water could be thought of as an energy source for the release of light from solid particles suspended in a cooling column of air above ground. But this theory could not explain the occurrence of light from the sky.

The occurrence of lights during earthquakes may be explained by the sunspot activities during solar maximum. The Earth has a magnetic field with north and south poles. The magnetic field of the earth is surrounded in a region called the magnetosphere. The magnetosphere prevents most of the particles from the sun, carried in solar wind from hitting the earth. Some particles from the solar wind can enter the magnetosphere. The particles that enter from the magnetotail travel toward the Earth and create the aurora oval light shows. Dangerous particles are not able to penetrate to the Earth's surface but are forced by the magnetic field to move around the Earth. Particles gain entry through the cusps that are shaped like funnels over the polar regions or they gain entry far downstream from the Earth. The particles that enter downstream travel toward the Earth and are accelerated into the high-latitude ionosphere and produce the auroral oval light shows. Since the most intense auroras occur at solar maximum, it was once thought that the Sun hurled material out during these raised times of solar activity and that that material funneled directly into the polar cusps. However, we now understand that the electrons that cause the auroras come in downstream or from the Earth's magnetic tail. These electrons that enter at the magnetotail are energized locally within the magnetosphere. The solar wind, emanating from the Sun, injects plasma into the magnetosphere and transfers energy to it. Several times a day, the magnetosphere undergoes a disturbance called a substorm. As the substorm grows, most of the solar wind energy is dissipated within the magnetosphere, ionosphere and upper atmosphere.

This disturbance ultimately causes auroral displays, the acceleration of charged particles to high energies, the emission of intense plasma waves and electromagnetic waves, and the generation of strong ionospheric currents that produce significant changes in the upper atmosphere. These waves and currents often result in severe problems on Earth with communications, power supplies, and spacecraft electronics.

Other higher energy particle radiation that could pose a danger to life here on Earth is forced to drift around the Earth within two large donut-shaped regions called the radiation belts. Invisible magnetic fields are the reason that particle radiation moves in this way. Before the occurrence of the catastrophic earthquake on 26th January 2001 at Kutch, IMAGE spacecraft captured an invisible magnetic tail at Gujarat, India. This is a major precursor of the earthquake. More advanced researches should be conducted to identify the geolatitude-longitude which is likely to be affected by the magnetic tail, which may trigger earthquakes in active fault areas. A sudden rise in electron flux and planetary indices 36 hrs before 26th January 2001 earthquake of Kutch, (Gujarat, India) and its sudden fall before the occurrence of earthquake was observed. Similar observations were made in several other cases also including the Andaman Islands. Coronal Mass Ejection, increase in Kp values (more than 4), sudden increase in Kp indices and electron flux can be forewarning of seismic disturbance in earthquake prone active fault areas and other environmental changes of earth. On 24th January 2001 an Earth directed coronal mass was ejected, which took two days to reach the earth's surface and may have triggered the active fault in the Katrol Formation of Kutch to produce a major earthquake of magnitude 7.9 in Gujarat, west coast of India. This area was reported as seismically active. In the entire world, a total of 65 earthquakes have been reported on the same day. Earth directed Coronal Mass Ejection produced a suspected invisible tail of electrified gas. IMAGE spacecraft spotted the tail, which streams from earth towards Sun. The region laced by earth's magnetic field, called the magnetosphere, dominates the behavior of electrically charged particles in space near earth and shields our planet from the solar wind. Explosive events on the Sun can charge the magnetosphere with energy, generating magnetic storms that occasionally may affect the active faults in igneous/sedimentary/metamorphic geosphere and change its viscosity to trigger the shallow focus earthquake. It may be interesting to observe the series of Earth directed Coronal Mass Ejections and the occurrence of earthquakes globally. It is not a specular correlation that an earthquake of 6.0 magnitude that occurred on 11th August 2003 at Andaman Island of Indian subcontinent was preceded by a rise in Kp indices, Electron flux as well as X-ray flux.

## 3. Conclusions

**Figure 2.**
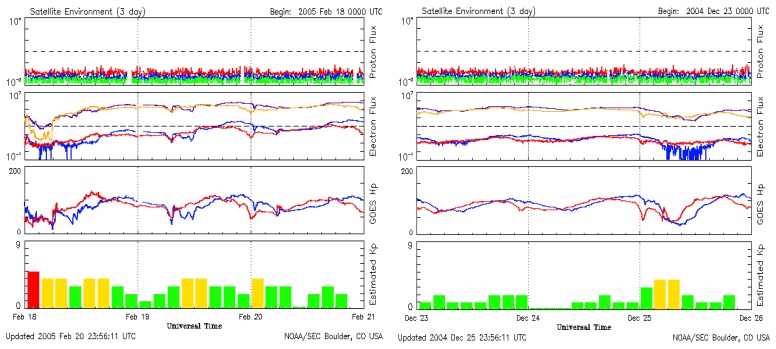
Influence of sunspots on Sun-Earth environment, showing low Kp and low **E-flux** before snowfall/rainfall, high Kp and high **E-flux** before an earthquake and very low Kp and low **E-flux** after an earthquake. Courtesy, SOHO/NASA.

It can be concluded that a sudden l drop in Kp, Electron flux, Proton flux and X-ray flux is an indication of atmospheric disturbance (Figure 2). This incidence may be followed by anomalous behavior of Indian monsoon, which ultimately leads to erratic rainfall pattern. Due to low Electron flux the local drop in temperature in the upper part of atmosphere leads to condense the clouds on the affected part of the earth. Contrary to this the rise in Kp indices, E-flux and Proton flux, as well as X-ray flux, leads to the occurrence of earthquakes. It is possible that the high particle flux on the earth is responsible for the release of more near infrared radiation before the earthquake as rocks rubbing together--is not responsible for the radiation field. The position of the Sunspot in the Sun is important; Coronal Mass Ejection from the sun may lead to catastrophic changes in a particular part of the planet Earth [36]. Starbursts are explosions triggered by intense magnetic field created by fast spinning, compact stellar corpses known as magnetar. Starbursts initiate the Sun to develop low Planetary Indices (Kp) and low Electron flux (E-flux) conditions of the Sun-Earth Environment. E-flux induces the variation of production of ionospheric currents. Ionospheric currents are produced by geomagnetic storms originating from the Star-Sun-Earth environment. Ionospheric current variation has direct influence on atmospheric temperature. On 25^th^ December 2004 and 23^rd^ February 2005 hailstorms and snowstorms were reported in the Northern Hemisphere, while in the tropics a sudden drop in temperature led to foggy and smoggy conditions. An earthquake measuring 9.0 of Richter scale on 26th December followed the sudden changes in the environment. An earthquake measuring 6.4 on the Richter scale occurred on Tuesday 22 February in Zarand, Kerman Province (750 KM south-east of Tehran) at 05:55 local time. Weather conditions in the region were difficult as there was heavy snowfall, resulting in a number of road blockades.

Here I show a theoretical correlation between Starbursts, Solar minimum, Sun-Earth environment and snowfall.

a) Significant changes in Star-Sun-Earth environment have been observed before the heaviest snowfall of the decade throughout the planet Earth. January and February are the snowiest months in the UK, whilst snowfall has also been recorded in March, April, May and June. The environment of the Earth is partially controlled by the heliophysical variables of the Sun. The solar variability is due to the perturbed nature of the solar core, and this variability is controlled by the variability of the solar neutrino flux. An interior dynamo exists in the core of the Sun, which is responsible for the solar activity cycle, further it is also responsible for E-flux and Kp values. The Stars further control the heliophysical variables of the Sun. Changes in cosmic-ray flux are the major reason for temperature changes over the past 500 million years. These changes are induced by the neutron star burst. It has been noticed in December 2004 as well as in February 2005 in Los Alamos National Laboratory that there were unexpected star explosions halfway across the galaxy. This type of explosion produces high-energy γ rays. This explosion packed so much power that it briefly altered Earth's upper atmosphere during December 2004 and February 2005. The blast originated about 50,000 light-years away and was detected on December 27. The blast was caused by magnetar. This star blast has influenced the geophysical variables of sun and sudden lowering in planetary indices Kp and E-flux. This might have been caused by repulsion of the magnetic field in Sun-Earth Environment and the magnetic field of star flare. Global warming in this century has corresponded with lowered cosmic ray intensities. Cosmic rays help the formation of dense clouds in the lower atmosphere while having a little negative effect on cloud cover in the upper atmosphere. The low clouds retain more surface energy, keeping the surrounding air hot, while the high clouds reflect more sunlight into space keeping the upper atmosphere cooler. Reconstruction of the solar magnetic field provides the major parameter needed to reconstruct the secular variation of the cosmic ray flux impinging on the terrestrial atmosphere since a stronger solar magnetic field more efficiently shields the earth from cosmic rays. Further it has been stated that cosmic rays affect the total cloud cover of the earth and thus drive the terrestrial climate. These point to a more rigorous and thorough study of the link between sun and climate change. Chandra X-ray observatory has seen X-ray outbursts from the star, which has helped to show that the magnetic field of the star is interacting with an orbiting disk of gas, and causing it to flare up intermittently. An attempt has been made in this paper to highlight the influence of star flares on Sun-Earth environment. Decrease in magnetic values and electron flux are also noticed after the earth directed star storm. These heliophysical and cosmological factors e.g. E-flux and Kp have direct relationships with the snowfall in higher latitudes and higher altitudes of lower latitudes in 25^th^ December 2004 and 22^nd^-23^rd^ February 2005. Movement of clouds, rainfall and development of fog and smog on lower altitudes of lower latitude has also been recorded on these days. Although the Sun is known to be a variable star, its total output of radiation is often assumed to be so stable and in general its' possible impact on climate is neglected. Testimony to this assumption is the term that has been employed for more than a century to describe the radiation in all wavelengths received from the Sun, the so-called solar constant, whose value at the mean Sun-Earth distance is a little over 1.37 kW / m² per unit of surface, but in truth, the solar constant varies. Earth's magnetospheric current systems are directly driven by the solar wind and are predictable in a rather deterministic sense 18. Variation in the intensity of galactic cosmic radiation observed on the ground from 1959 to 2000, (Figure 1) has been compared with that of the index of sunspots (dotted line). The extra galactic cosmic rays are responsible for the ionization in the lower atmosphere below 35 km and are the principal source of ionization over the oceans.
b) The permanent component of cosmic radiation comes from the galaxy. It consists of very highly charged particles ejected by the gigantic explosions of supernova, massive stars that have reached the end of their days. These particles are atoms, which have been stripped of their electrons because of the temperatures within these giant stars. They are of different types, primarily hydrogen nuclei (protons) and helium nuclei (alpha particles), but also there are heavier nuclei such as iron and nickel. They travel at close to the speed of light. We see that during periods of high solar activity, the cosmic radiation is less intense, as the cosmic effect suppresses the formation of sunspots. During the impact of cosmic rays with the atmosphere of the Earth, ionization takes place. During this ionization the thermal energy is utilized to produce a regional fall in the temperature of the earth, which may lead to sudden snowfall in higher latitude and altitudes of the Earth.

**Figure 3.**
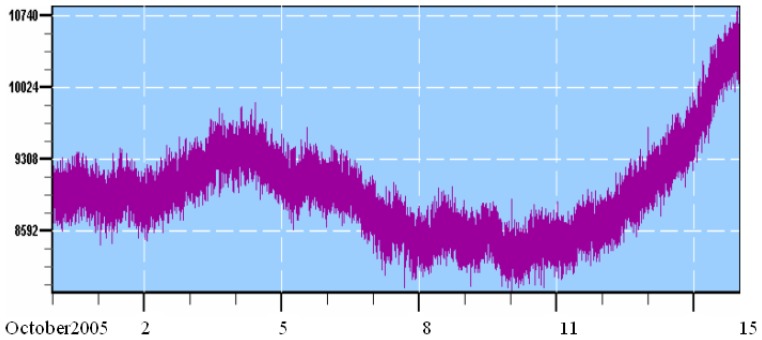
Cosmic ray variation before snowfall and subsequent earthquake on 8th October 2005, establishing forebursh effect and its relation with environment of the Earth (shown in between 8000 to 8100 as low during earthquake and 10740 as high after earthquake).

The magnetic field around the Earth protects the planet from cosmic rays. This field is stronger when the Sun is more active i.e., emitting more ultraviolet radiation and displaying more sunspots hence fewer cosmic rays can penetrate Earths atmosphere. A direct influence of cosmic rays with the fall in temperature of whole world was observed during the years of starburst (Table 2). A correlation between the global average of low cloud cover and the flux of Galactic cosmic Rays incident in the atmosphere has been observed (Figure 3). Using data derived from International Satellite Cloud Climatology Project (ISCCP) and ground based diffuse solar radiation data provide a new evidence for a non linear effect of galactic cosmic rays on environment of the earth [36]. The observation was established across the UK, on days of high cosmic ray flux (above 3600×10^2^ neutron counts h^-1^) compared to low cosmic ray flux, the chance of an overcast day increases by 20%. They also observed that during Forbursh events simultaneous decreases occur in the diffuse fraction. Cosmic ray variations are possible to correlate with geomagnetic storms, which changes the ionospheric currents and triggering of seismic activities [37, 38 and 39]. For hailstorm or snowstorm or heavy cloud formation it is essential that Earth's atmosphere should have a sudden increase in micro dust particles. Data of star storm shows that recent hailstorm has developed subsequently after the star storm. The reason for the weakening of the Sun's magnetic shield is the increased solar activity, which leads to a highly disordered field configuration. In the mid-1990's, during the last solar minimum, the Sun's magnetic field resembled a dipole field with well-defined magnetic poles (North positive, South negative), very much like the Earth. Unlike Earth, however, the Sun reverses its magnetic polarity every 11 years. The reversal always occurs during solar maximum. That's when the magnetic field is highly disordered, allowing more interstellar dust to enter the Solar System. It is interesting to note that in the reversed configuration after the recent solar maximum (North negative, south positive), the interstellar dust is even channeled more efficiently towards the inner Solar System. It was expected that more interstellar dust would occur from 2005 onwards, but it has appeared in December 2004. The Sun has entered the zodiac's 13th house. An interstellar wind hit our planet. It's a helium-rich breeze from the stars, flowing into the solar system from the direction of Ophiuchus. The Sun's gravity focuses the material into a cone and Earth passes through it during the first week of December. Earth was inside the cone during 25th December 2004 and 23rd February 2005. Grains of stardust are very small, about one hundredth the diameter of a human hair, hence they do not directly influence the planets of the Solar System. However, the dust particles move very fast, and produce large numbers of fragments when they impact asteroids or comets. It is, therefore, conceivable that an increase in the amount of interstellar dust in the Solar System will create more cosmic dust by collisions with asteroids and comets. It is possible that the increase of stardust in the Solar System will influence the amount of extraterrestrial material that rains down to Earth. How the Earth's surface temperature adjusts to a given change in star-solar radiation depends on the processes by which the climate system responds to variations in the energy it receives [40, 41, 42, 43 and 44]. Some of these factors amplify the effects of changes that are imposed; others reduce them. Lumped together, they make up what is called the sensitivity of the climate system, which indicates the number of degrees by which the mean-surface temperature will be raised or lowered in response to a given change, up or down, in solar and/or extra terrestrial radiation or any other climate driver. The temperature data of west USA shows a declining trend of -0.68 degree F /decade, which is considered to be anomalous in the rising trend of 0.05 deg F /decade for USA.

Thus, it is concluded that starbursts influences variables of the Sun-Earth environment, which in turn leads to climate change including snowfall. Sudden change in these variables like Kp, Proton flux and E-flux influence environment of the Earth abruptly (Figure 2). If E-flux hike is responsible for global warming then E-flux lowering may lead to snowfall. On 22nd December 2004 and 22nd -23rd February 2005 sudden fall in electron flux and Kp indices was recorded by SOHO satellite. Widespread snowfall was recorded in United Kingdom and other parts of the world on 25th December 2005 and further on 23rd February 2005 (Table 1). Further the rise of E-flux leads to normalize the condition. The influence of the starburst might have influenced the E-flux and thus leading to snowfall on 25th December 2004 and 22nd and 23rd February 2005. The cosmic influence on the Sun is so intense that until March 2005 the number of sunspots was far fewer and the E-flux, as well as Kp indices, was also very low. This might be the possible reason for this years extended snowfall and cold wave. The influence of Star on the Sun-Earth environment is being reported theoretically first time. Regular monitoring of Star-burst and its influence on Sun-Earth environment may lead to more accurate environmental prediction.

## Figures and Tables

**Table 1.** Sudden snowfall and rainfall after the rise in temperature in higher altitude and latitude on 25th December 2004 before the tsunami and earthquake in Indonesia. This observation has been recorded across the world including Europe, Asia and USA. http://news.bbc.co.uk/2/hi/uk_news/4124715.stm#

**Location**	**Snowfall/Cold wave**	**Anomalous**	**Remarks**

Liverpool (UK)	Snowfall	Yes	Low Kp and Low E-flux http://www.metoffice.gov.uk
Birmingham (UK)	Snowfall	Yes	Low Kp and Low E-flux http://www.metoffice.gov.uk
Manchester (UK)	Snowfall	Yes	Low Kp and Low E-flux http://www.metoffice.gov.uk
Cardiff (UK)	Snowfall	Yes	Low Kp and Low E-flux http://www.metoffice.gov.uk
Aberdeen (UK)	Snowfall	Yes	Low Kp and Low E-flux http://www.metoffice.gov.uk
Belfast	Snowfall	Yes	Low Kp and Low E-flux http://www.metoffice.gov.uk
Crosby	Snowfall	Yes	Low Kp and Low E-flux http://www.metoffice.gov.uk
Woodford	Snowfall	Yes	Low Kp and Low E-flux http://www.metoffice.gov.uk
Houghton, Michigan USA	Anomalous Snowfall	Yes	Low Kp and Low E-flux 4–5 inches of fresh snow http://www.americanoakresort.com/trailold2004.htm
Louisiana, Texas, USA	Very high Snowfall	Yes	Low Kp and Low E-flux http://en.wikipedia.org/wiki/2004_Christmas_Eve_Snowstorm
Galveston, Texas, USA	Very high Snowfall	Yes	Low Kp and Low E-flux http://www.hprcc.unl.edu/nebraska/december25–2004.html Portions of South Texas have their first White Christmas ever as snow amounts measured from 1.5” in Brownsville to over one foot in Victoria
New York, USA	Very high Snowfall	Yes	Low Kp and Low E-flux http://en.wikipedia.org/wiki/2004_Christmas_Eve_Snowstorm
Queensland, Australia	Cyclone with cold wave	Yes	Low Kp and Low E-flux http://en.wikipedia.org/wiki/2004
Jammu Kashmir, Shimla, India	Snowfall with cold wave	Yes	Low Kp and Low E-flux http://en.wikipedia.org/wiki/December_2004#December_25.2C_2004

Source: Meteorological Office United Kingdom, BBC weather News UK and NOAA USA.

**Table 2.** Starbursts lower the temperature of earth. Forbursh effect lowers the **E-flux** from Sun followed by a fall in atmospheric temperature. Source: NCDC USA and Messier catalog.

S.No.	Starburst year	Star ID number	Anomalous fall in temperature

1	1885	M31	Yes
2	1909	M101	Yes
3.	1914	M100	Yes
4.	1923	M83	Yes
5.	1926	M61	Yes
6.	1945	M83	Yes
7.	1950	M83	Yes
8.	1967	M99	Yes
9.	1969	M49	Yes
10.	1972	M99	Yes
11.	1983	M83	Yes
12.	1986	M99	Yes
13.	1988	M58	Yes
14.	1989	M58	Yes
15.	1989	M66	Yes
16.	1999	M88	Yes
17.	2004	M60, V838	Yes
